# External validation of the Ruptured Arteriovenous Malformation Grading Scale (RAGS) in a multicenter adult cohort

**DOI:** 10.1007/s00701-022-05433-1

**Published:** 2022-12-07

**Authors:** Lukasz Antkowiak, Marta Rogalska, Piotr Stogowski, Placido Bruzzaniti, Pietro Familiari, Magdalena Rybaczek, Tomasz Klepinowski, Weronika Grzyb, Mikolaj Zimny, Mateusz Weclewicz, Anna Kasperczuk, Wojciech Kloc, Adam Rudnik, Leszek Sagan, Tomasz Lyson, Zenon Mariak, Antonio Santoro, Marek Mandera

**Affiliations:** 1 Department of Pediatric Neurosurgery, Medical University of Silesia, Katowice, Poland; 2 Faculty of Medicine, Medical University of Warsaw, Warsaw, Poland; 3 Department of Neurosurgery, Copernicus Hospital, Gdansk, Poland; 4 Department of Neurosurgery, Spaziani Hospital, Frosinone, Italy; 5 Department of Human Neurosciences, Sapienza University, Rome, Italy; 6 Department of Neurosurgery, Medical University of Bialystok, Bialystok, Poland; 7 Department of Neurosurgery, Pomeranian Medical University Hospital No. 1, Szczecin, Poland; 8 Department of Neurosurgery, Medical University of Silesia, Katowice, Poland; 9 Faculty of Mechanical Engineering, Institute of Biomedical Engineering, Bialystok University of Technology, Bialystok, Poland; 10 Department of Psychology and Sociology of Health and Public Health, School of Public Health, Collegium Medicum, University of Warmia–Mazury in Olsztyn, Olsztyn, Poland

**Keywords:** Brain arteriovenous malformations, Vascular malformations, AVM rupture, Intracerebral hemorrhage, Prognosis

## Abstract

**Purpose:**

While Ruptured Arteriovenous Malformation Grading Scale (RAGS) has recently been validated in children, the literature lacks validation on adults exclusively. Therefore, we aimed to determine the validity of RAGS on the external multicenter adult cohort and compare its accuracy with other scales.

**Methods:**

A retrospective analysis was performed in five neurosurgical departments to extract patients who presented with the first episode of acute brain arteriovenous malformation (bAVM) rupture between 2012 and 2019. Standard logistic regression and area under the receiver operating curve (AUROC) calculations were performed to determine the value of the following scales: intracerebral hemorrhage (ICH), AVM-associated ICH (AVICH), Spetzler-Martin (SM), Supplemented SM (Supp-SM), Hunt and Hess (HH), Glasgow Coma Scale (GCS), World Federation of Neurological Surgeons (WFNS), and RAGS to predict change in categorical and dichotomized modified Rankin Scale (mRS) across three follow-up periods: within the 6 months, 6 months to 1 year, and above 1 year.

**Results:**

Sixty-one individuals with a mean age of 43.6 years were included. The RAGS outperformed other grading scales during all follow-up time frames. It showed AUROC of 0.78, 0.74, and 0.71 at the first 6 months, between 6 and 12 months, and after 12 months of follow-up, respectively, when categorized mRS was applied, while corresponding values were 0.79, 0.76, and 0.73 for dichotomized mRS, respectively.

**Conclusion:**

The RAGS constitutes a reliable scale predicting clinical outcomes following bAVM rupture among adults. Furthermore, the RAGS proved its generalizability across medical centers with varying treatment preferences.

## Introduction

Although a brain arteriovenous malformation (bAVM) rupture accounts for only 2% of hemorrhagic strokes, up to 50% of patients with diagnosed bAVM initially present with bleeding [1, 2]. Specifically, in the subpopulation of young adults, bAVM rupture constitutes an important cause of hemorrhagic strokes, significantly affecting patients' quality of life, resulting in a 13% mortality and a prominent 40% disability rate [9, 16]. Hence, the ability to precisely predict patient outcomes at the early stage of the hospital stay would be desirable.

The therapeutic approach to ruptured bAVMs should be focused on preventing future rebleeding while being weighed against the risk associated with invasive treatment [3]. Thus, a precise evaluation of the patient’s prognosis could aid in the decision-making process regarding the optimal management strategy. Routinely, the patient outcome has been forecasted using general clinical status assessment tools, including Glasgow Coma Scale (GCS) [15], Hunt and Hess (HH) scale [7], World Federation of Neurological Surgeons (WFNS) scale [11], or Intracerebral hemorrhage (ICH) score [5]. Nonetheless, none of them was initially designed to determine the prognosis of patients with ruptured bAVMs. In 2016, Neidert et al. [10] proposed an AVICH (AVM-associated ICH). Although the authors made a step toward estimating prognosis in patients with ruptured bAVMs, their scale can be applied only in the event of the accompanying ICH, which is not the case in roughly 20% of ruptured bAVMs [12]. In 2020, Silva et al. [13] introduced the Ruptured Arteriovenous Malformation Grading Scale (RAGS), which was intended to predict clinical outcomes following bAVM rupture specifically (Table 1). In addition to its high accuracy in outcome prognostication, it does not depend on the therapeutic approach and remains relatively simple. It constitutes an extension of the HH scale, with additional evaluation of the patient’s age, the presence of deep venous drainage, and the eloquent bAVM location. The recent single-center study by Garcia et al. [4] proved the validity of the RAGS in the external pediatric cohort. However, the literature lacks RAGS validation conducted solely on adults. Therefore, our study aimed to determine the validity of RAGS on the external multicenter cohort of adult patients and compare its predictive accuracy with previously applied scales.

**Table 1:** The RAGS [13]

Variable	Value	Number of points
HH score	1–5	1–5
Age	< 35	0
	35–70	1
	> 70	2
Deep venous drainage	No	0
	Yes	1
Eloquent	No	0
	Yes	1
		Range 1–9

*HH*, Hunt and Hess; *RAGS*, Ruptured Arteriovenous Malformation Grading Scale

## Material and methods

### Study cohort

A retrospective analysis of the medical charts of adult patients, who presented with bAVM rupture between 2012 and 2019, was performed. The following medical centers were involved: (1) Medical University of Silesia in Katowice; (2) Copernicus Medical Center in Gdansk; (3) Pomeranian Medical University in Szczecin; (4) Medical University of Bialystok; (5) Sapienza University of Rome. The bAVM rupture was defined as an acute onset of headache, seizure, or neurological deficit accompanied by an acute hemorrhage on head CT/MRI scans.

### Inclusion and exclusion criteria

Only adults (aged > 18 years on admission) who presented with the first episode of acute bAVM rupture, had a full set of clinical and radiological data, and completed at least 13 months of follow-up after the initial presentation, were included. A history of previous bAVM rupture or its treatment, lacking clinical or radiological data, and incomplete follow-up data constituted exclusion criteria.

### Data extraction

Medical charts of all included individuals were thoroughly reviewed to extract patient demographics, detailed initial clinical presentation, and neurological deficits. For the purpose of RAGS validation, the degree of disability before bAVM rupture was retrospectively determined using the modified Rankin Scale (mRS). Based on the digital subtraction angiography (DSA) data, the following bAVM features were determined: bAVM location, nidus size, a pattern of venous drainage, deep perforating artery supply, associated feeding artery aneurysm, the presence and volume of intraparenchymal hemorrhage (IPH), signs of intraventricular hemorrhage (IVH), and the presence of subarachnoid hemorrhage (SAH). For each patient, the decision on the preferred therapeutic approach was made by the interdisciplinary team consisting of a neurosurgeon, neuroradiologist, and radiotherapist, when necessary. All patients underwent further clinical follow-up after discharge at three follow-up periods: within 6 months, from 6 months to 1 year, and above 1 year, with a precise assessment of the degree of disability using mRS. Since patients presented in unstandardized follow-up points, the follow-up was divided into the abovementioned time ranges.

Based on the detailed medical documentation, each individual was evaluated using the HH scale [7], GCS [15], ICH scale [5], WFNS scale [11], AVICH scale [10], Spetzler-Martin (SM) scale [14], Supplemented SM (Supp-SM) scale [8], and RAGS at admission.

### Statistical analysis

In order to determine each scale’s (AVICH, ICH, SM, Supp-SM, HH, GCS, WFNS, and RAGS) accuracy in predicting clinical outcomes after bAVM rupture, the values of each abovementioned scale calculated at admission were correlated with the increase of the mRS score at three follow-up periods: within the 6 months, from 6 months to 1 year, and above 1 year, in reference to the pre-rupture patient’s mRS score. Standard logistic regression and area under the receiver operating curve (AUROC) calculations were performed for categorical mRS change (0–6) and dichotomized mRS change (divided into favorable (mRS 0–2) and unfavorable (mRS 3–6) scores) in order to determine the accuracy of each scale to predict clinical outcomes. The AUROC of 0.5 indicated the scale’s inability to differentiate results, whereas the value of 1.0 reflected perfect discrimination. Statistical analysis were performed using Statistica 13.3 (StatSoft Polska, Krakow, Poland) and PQStat 1.8.4 (PQStat Software, Poznan, Poland) software.

## Results

### Study cohort

A total of 61 consecutive individuals who presented with bAVM rupture were included in the study. There were 38 female (62.3%) and 23 male (37.7%) patients, with a mean age of 43.6 years (SD; 16.9 years). Detailed patient characteristics are presented in Table 2.

**Table 2:** Patient characteristics

Feature	Value
Number of patients	61
Mean age (SD)	43.6 (16.9)
Sex—female, *n* (%)	38 (62.3)
HH score, *n* (%)	
1	22 (36.1)
2	21 (34.4)
3	13 (21.3)
4	2 (3.3)
5	3 (4.9)
SM grade, *n* (%)	
1	14 (23)
2	24 (39.3)
3	18 (29.5)
4	3 (4.9)
5	2 (3.3)
Mean nidus size in cm (SD)	2.3 (1.6)
Left side, *n* (%)	26 (42.6)
Right side, *n* (%)	34 (55.7)
Midline, *n* (%)	1 (1.6)
Supratentorial, *n* (%)	55 (90.2)
Infratentorial, *n* (%)	6 (9.8)
Eloquent area, *n* (%)	29 (47.5)
Deep venous drainage, *n* (%)	22 (36.1)
Diffuse nidus, *n* (%)	19 (31.2)
Feeding artery aneurysm, *n* (%)	6 (9.8)
Concurrent aneurysm, *n* (%)	10 (16.4)
IPH, *n* (%)	49 (80.3)
IVH, *n* (%)	15 (24.6)
Deep perforating artery supply, *n* (%)	17 (27.9)
Mean time from presentation to the bAVM therapy in days (SD)	11.7 (23.9)
Surgery, *n* (%)	15 (24.6)
Embolization only, *n* (%)	28 (45.9)
Multimodal treatment, *n* (%)	18 (29.5)
Rate of cure, *n* (%)	43 (70.5)
Mean follow-up in years (SD)	3.73 (23.1)
Re-rupture, *n* (%)	3 (4.9)
Final mRS, *n* (%)	
0	10 (16.4)
1	29 (47.5)
2	17 (27.9)
3	2 (3.3)
4	0
5	1 (1.6)
6	2 (3.3)

*HH*, Hunt and Hess; *SM*, Spetzler-Martin; *IPH*, intraparenchymal hemorrhage; *IVH*, intraventricular hemorrhage; *mRS*, modified Rankin Scale; *SD*, standard deviation; *bAVM*, brain arteriovenous malformation

The mean bAVM size was 2.3 cm, with the majority of lesions classified as SM II grade (39.3%), followed by SM III (29.5%), SM I (23%), SM IV (4.9%), and SM V (3.3%). All patients in our cohort underwent interventional bAVM treatment. Surgical resection as the solitary management constituted the initial modality in 15 patients (24.6%), 28 patients underwent only embolization (45.9%), while 18 individuals underwent multimodal treatment (the combination of at least 2 treatment modalities) (29.5%) (Table 2).

### RAGS evaluation

**Fig. 1:**
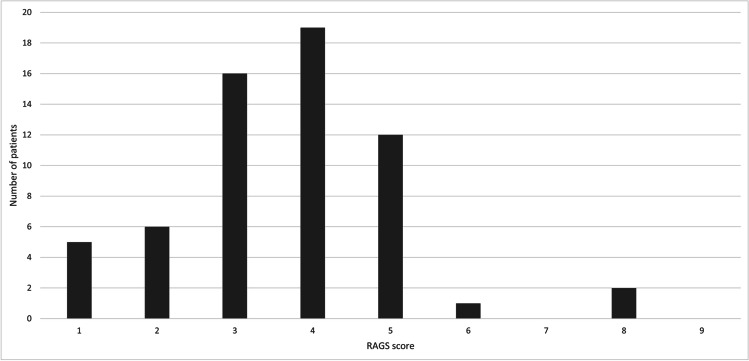
The Ruptured Arteriovenous Malformation Grading Scale (RAGS) score distribution within the study cohort

There were 5 patients with a RAGS score of 1 (8.2%), 6 patients with RAGS score of 2 (9.8%), 16 patients with RAGS score of 3 (26.2%), 19 patients with RAGS score of 4 (31.2%), 12 patients with RAGS score of 5 (19.7%), 1 patient with RAGS score of 6 (1.6%), and 2 patients with RAGS score of 8 (3.3%). We did not report any patients with RAGS scores of 7 and 9 (Fig. 1).

Through the AUROC analysis, we found that RAGS outperformed other grading scales (AVICH, ICH, SM, Supp-SM, HH, GCS, WFNS) both for categorical and dichotomized mRS during the entire follow-up period (Table 3). The RAGS showed AUROC of 0.78, 0.74, and 0.71 at the first 6 months, between 6 and 12 months, and after 12 months of follow-up, respectively, when categorized mRS was applied, while the corresponding values were 0.79, 0.76, and 0.73 for dichotomized mRS.

**Table 3:** Summary of the AUROC values for each grading system to predict clinical outcome at three distinct time periods

	Categorical mRS (0–6)			Dichotomized mRS (0–2 vs 3–6)		
	First 6 months	6–12 months	Last follow-up (> 12 months)	First 6 months	6–12 months	Last follow-up (> 12 months)
RAGS	**0.78**	**0.74**	**0.71**	**0.79**	**0.76**	**0.73**
AVICH	0.64	0.61	0.61	0.65	0.63	0.64
ICH	0.67	0.55	0.53	0.68	0.57	0.56
SM	0.54	0.60	0.48	0.55	0.62	0.51
Supp-SM	0.54	0.59	0.60	0.55	0.61	0.63
HH	0.71	0.68	0.67	0.72	0.70	0.70
GCS	0.71	0.70	0.69	0.72	0.72	0.72
WFNS	0.64	0.68	0.64	0.65	0.70	0.67

*AUROC*, area under the receiver operating characteristic; *mRS*, modified Rankin Scale; *RAGS*, ruptured arteriovenous malformation grading scale; *AVICH*, AVM-associated ICH; *ICH*, Intracerebral hemorrhage; *SM*, Spetzler-Martin; *Supp-SM*, Supplemented Spetzler-Martin; *HH*, Hunt and Hess; *GCS*, Glasgow Coma Scale; *WFNS*, World Federation of Neurological Surgeons

## Discussion

Since the publication of the RAGS in 2020 [13], it has been externally validated by Garcia et al. [4] in the pediatric cohort, representing the part of Silva et al. [13] cohort below 35 years of age. The novelty of our study is associated with the inclusion of solely adult patients (above 18 years of age), thus filling the previously existing gap in the literature considering the validation of RAGS on an external adult cohort.

We found that RAGS outperformed multiple previously applied scales, showing the highest AUROC of 0.79 for the dichotomized, and 0.78 for the categorical mRS score evaluated within the first 6 months of follow-up. Additionally, our analysis revealed that AUROC values were higher for dichotomized than for categorical mRS score in all assessed time periods during follow-up. Similar findings were reported in Silva et al. study [13], although the authors highlighted the importance of categorical mRS score in capturing nuances in patient clinical status. Therefore, we presume that despite being the most accurate scale in predicting patient outcomes following bAVM rupture, RAGS is less accurate in expressing slight differences in patient recovery than it is in distinguishing between favorable and unfavorable outcomes.

Contrarily to Garcia et al. study [4], which compared RAGS to merely two other scales (HH and GCS), we performed an extensive comparison between RAGS and seven other routinely applied scales (AVICH, ICH, SM, Supp-SM, HH, GCS, WFNS), similarly to the original Silva et al. paper [13]. Additionally, in Silva et al. [13] study, the ICH score outperformed RAGS concerning the categorical mRS in the follow-up period between 9 and 12 months. Moreover, the AUROC values for the ICH score were noticeably similar to the AUROC values for the RAGS score, especially when categorical mRS was applied [13]. Contrarily, our study demonstrated the indisputable superiority of RAGS, which outperformed even the ICH score.

Both Silva et al. [13] and Garcia et al. [4] reported the highest AUROC values for RAGS, amounting to 0.86 and 0.82, respectively, whereas our analysis demonstrated the highest AUROC value of 0.79. However, in contrast to the abovementioned papers, our study was based on the multicenter cohort with various preferred treatment modalities in each institution. Although RAGS can be applied irrespective of the treatment approach, the variability of treatment preferences in our cohort could potentially lower the AUROC of RAGS compared to the analysis based on data from a single institution. Notably, Silva et al. [13] reported the highest AUROC values for RAGS, with 76% of patients treated surgically. In Garcia et al. study [4], slightly lower AUROC values for RAGS were achieved, with 31% of patients treated by means of bAVM excision. In turn, we reported relatively lowest AUROC values, with 24.6% of individuals undergoing surgery. Although we stress that RAGS applicability does not depend on the chosen treatment modality, its original implementation on the mainly surgically treated cohort might implicate its higher accuracy in predicting outcomes in surgical patients than in individuals treated by means of other modalities.

On the other hand, despite the high percentage of low-grade bAVMs in our cohort, most patients underwent exclusively endovascular treatment, which does not necessarily represent the current first-line standard of care [6, 17]. Moreover, despite the dissimilarities in the preferred management modality between our study and Silva et al. paper [13], RAGS remained the most accurate scale in the prognosis assessment, which additionally confirms its independence from the selected treatment method. Moreover, the discrepancies in the percentage distribution of applied management techniques between all three cohorts, with the preserved highest AUROC for RAGS, further support the thesis of RAGS generalizability. However, future multicenter studies with larger patient cohorts are needed to confirm this conclusion unequivocally. It would also be of great interest to externally validate RAGS accuracy in predicting outcomes among patients mainly with high-grade bAVMs. Additionally, the invariant simplicity and improved accuracy of RAGS compared to currently applied scales render it an efficient grading system that is easy to implement into routine clinical practice.

Traditionally, the selection of an appropriate management strategy depends on AVM characteristics and overall patient condition. The identification of patient’s prohibitive neurological status might be facilitated using RAGS. Although we stress that the RAGS score should not be utilized as a surgical decision-making tool, it provides insight into the patient’s prognosis and potential for recovery after bAVM rupture. Especially in borderline cases, a more positive forecasted outcome might encourage a more aggressive interventional approach. Furthermore, as previously stated by Silva et al. [13], this grading system might help surgeons confront the operative results with initially anticipated outcomes. For instance, a poor outcome after low SM-grade bAVM surgery might be attributed to the initially poor prognosis (high RAGS score) or classified as an unanticipated perioperative complication (low RAGS score) [13].

Furthermore, we determined the score's validity throughout the follow-up period (3.76 years), similar to the one in the original RAGS paper (4 years [13]) and in Garcia et al. study (3.9 years [4]), which demonstrates its usefulness as a long-term prognostic tool and additionally favors its application in routine clinical settings.

### Limitations

Despite the multicenter design, our research was based on a limited cohort, which might have influenced the external validity of our findings. However, in order to achieve the homogeneity of our results, precise inclusion criteria pursued the exclusion of many patients who did not comply with the standards of this study. Moreover, the retrospective character of our study might have created a bias resulting from retrograde data evaluation. Further prospective, multicenter collaboration performed on a larger cohort would be desirable to unequivocally determine the validity of the RAGS scale to reflect clinical outcomes.

## Conclusions

Through the external multicenter adult cohort, we found that RAGS outperformed other scales frequently implemented to evaluate patients with bAVM rupture. However, the accuracy of RAGS appears to be lower in distinguishing slight differences in patient recovery than in expressing discrepancies between favorable and unfavorable outcomes. Since our study was based on the multicenter experience, the RAGS proved its generalizability across medical centers with varying treatment preferences.
